# Long-acting reversible contraceptives use among adolescent girls and young women in high fertility countries in sub-Saharan Africa

**DOI:** 10.1186/s12978-022-01494-8

**Published:** 2022-11-16

**Authors:** Francis Sambah, Richard Gyan Aboagye, Abdul-Aziz Seidu, Charles Lwanga Tengan, Tarif Salihu, Bright Opoku Ahinkorah

**Affiliations:** 1 College of Public Health, Medical and Veterinary Sciences, James Cook University, Townsville, QLD 4811 Australia; 2 Department of Health, Physical Education and Recreation, University of Cape Coast, Cape Coast, Ghana; 3 Department of Family and Community Health, Fred N. Binka School of Public Health, University of Health and Allied Sciences, Hohoe, Ghana; 4 Centre for Gender and Advocacy, Takoradi Technical University, Takoradi, Ghana; 5 Kumasi Centre for Collaborative Research in Tropical Medicince, Kwame Nkrumah University of Science and Technology, Kumasi, Ghana; 6 Department of Population and Health, University of Cape Coast, Cape Coast, Ghana; 7 School of Public Health, Faculty of Health, University of Technology Sydney, Sydney, Australia

**Keywords:** Predictors, Long-acting reversible, Contraceptive, Sub-Saharan Africa, DHS

## Abstract

**Background:**

Given the instrumental role long-acting reversible contraceptives (LARCs) play in reducing unintended pregnancies, there is a need to understand the factors that predict their use among adolescent girls and young women in high fertility countries. Our study examined the prevalence and predictors of LARCs use among adolescent girls and young women in high fertility countries in sub-Saharan Africa.

**Materials and methods:**

We pooled data from the women’s files of the most recent Demographic and Health Surveys (DHS) from 2010 to 2020 of the top ten high fertility countries in sub-Saharan Africa, which are part of the DHS programme. The total sample was 5854 sexually active adolescent girls and young women aged 15–24 who were using modern contraceptives at the time of the survey. Descriptive and multilevel logistic regression models were used in the analyses. The results were presented using percentages and adjusted odds ratio (AOR) with their respective 95% confidence intervals (CIs).

**Results:**

At the descriptive level, the overall prevalence of *LARCs* utilisation was 17.6% in the ten countries, with the lowest of 1.7% in Angola and the highest of 55.8% in Mali. Adolescent girls and young women who were married had a lower likelihood of *LARCs* utilisation than those who were never married [AOR = 0.63, 95% CI = 0.45, 0.88]. Adolescent girls and young women who wanted no more children had higher odds of LARCs use compared to those who wanted more children [AOR = 1.56, 95% CI = 1.09, 2.26]. Adolescent girls and young women with one to three births [AOR = 6.42, 95% CI = 4.27, 9.67], and those with four or more births [AOR = 7.02, 95% CI = 3.88, 12.67] were more likely to use LARCs compared to those who had no children. Countries in sub-Saharan Africa with lower probability of utilizing LARCs were Angola, Niger and Mozambique, whereas adolescent girls and young women in Mali had higher probability of utilizing LARCs.

**Conclusion:**

Our findings suggest that LARCs utilisation among adolescent girls and young women is low in high fertility countries in sub-Saharan Africa. To reduce the rates of unplanned pregnancies and induced abortions, it is imperative that adolescent girls and young women in sub-Saharan Africa are educated on the advantages of utilising LARCs. Additionally, governments, policymakers, and stakeholders in sub-Saharan Africa should raise awareness by executing health promotion measures to enhance the demand for LARCs among adolescent girls and young women. Achieving these would not only prevent unplanned pregnancies and induced abortions, but also help meet the United Nation’s health and well being for all as enshrined in Sustainable Development Goals 3 and 5.

## Plain language summary

The use of long-acting reversible contraceptives can contribute to the reduction of unintended pregnancies. Hence, knowledge of the prevalence and predictors of *long-acting reversible contraceptives* use among adolescent girls and young women in high fertility countries in sub-Saharan Africa is important in public health. Our study examined the predictors of long-acting reversible contraceptives among adolescent girls and young women in ten high fertility countries in sub-Saharan Africa. A sample of 5854 sexually active adolescent girls and young women were included in the study.

The overall prevalence of long-acting reversible contraceptives utilisation was 17.6% in the ten countries, with the lowest of 1.7% in Angola and the highest of 55.8% in Mali. Adolescent girls and young women who were married had a lower likelihood of using long-acting reversible contraceptives than those who were never married. Adolescent girls and young women who wanted no more children had higher odds of long-acting reversible contraceptive use compared to those who wanted more children. Adolescent girls and young women with one to three births, and those with four or more births were more likely to use long-acting reversible contraceptives than those who had no child. To reduce the rates of unplanned pregnancies and induced abortions, there is the need to educate adolescent girls and young women on the advantages of utilising *long-acting reversible contraceptives*. Additionally, governments, policymakers, and stakeholders in sub-Saharan Africa should raise awareness by executing health promotion measures to enhance the demand for long-acting reversible contraceptives among adolescent girls and young women.

## Background

Unintended pregnancy is a major problem among sexually active women and can result from incorrect, inconsistent, or non-use of contraception, or contraceptive failure—that is, becoming pregnant while using a family planning method [1]. There have been significant global efforts to reduce fertility rates and unplanned pregnancies [2]. Notwithstanding the increasing contraceptive availability, unplanned pregnancy remains a worldwide problem, representing as many as 30% of all known pregnancies [1]. Various approaches have been suggested to reverse this alarming trend, especially through the increased use of long-acting reversible contraceptives (LARCs) [1]. The World Health Organization (WHO) describes adolecents and young people as individuals aged 10–19 and 15–24 respectively. Adolescent pregnancy is a persistent global health problem [3]. Adolescent child birth account for 11% of all births worldwide, and in low- and middle-income countries (LMICs), complications from pregnancy, childbirth, and unsafe abortion are among the leading causes of morbidity and mortality among adolescent girls and young women [4].

The occurrence of adolescent childbearing is complex and far reaching, having effect not only on the adolescents but also on their children and their community. The occurrence and public health effects of adolescent pregnancy mirrors complex structural social problems and an unmet need for acceptable and effective methods in population. In 2006–2010, 82% of adolescents at risk of unintended pregnancy were currently using contraception, but only 59% used a highly effective method, including any hormonal method or intrauterine device. LARCs have higher efficacy, higher continuation rates and higher satisfaction rates compared with short acting contraceptives among adolescents who choose to use them [5]. Complications from intrauterine devices and contraceptive implants have been found to be less for adolescent girls and young women, which makes these methods safe for adolescents [5].

In six decades since 1950, fertility has dropped considerably in LMICs. Even so, high fertility which is defined as five or more births per woman over the reproductive career, characterizes 33 countries [6]. Twenty-nine of these countries are in sub-Saharan Africa (SSA). High fertility poses health risks for children and their mothers, reduces human capital investment, slows economic growth, and exacerbates environmental threats [7]. According to the Population Reference Bureau (PRB), Niger has the highest total fertility rate (7.1 average births for each woman), followed by Mali (6.3) and the Democratic Republic of the Congo (6.2). Some of the lowest total fertility rates are in South Korea (0.9), Taiwan (1.0) and Singapore (1.1) [8].

In 2010–2015, 8% of the global population lived in countries where women were having, on average, more than 5 births over a lifetime [9]. The safety, efficacy, and long-term cost–benefit factors of LARC use in the adolescent population have been well established [10]. However, only 24.7% of adolescent girls and young women in SSA use modern contraceptives, which include *LARCs* [11]. Age, marital status, religion, employment status, parity, exposure to mass media, desire for more children, ideal number of children and age at first sex have been identified as predictors of modern contraceptive use among adolescent girls and young women aged 15–24 in SSA [11].

Extensive studies have been conducted on LARC use among adolescent girls [12–14]. However, there is limited literature on predictors of *LARCs* use among adolescent girls and young women in high fertility countries especially those within SSA. Given the instrumental role LARCs play in cutting down unintended pregnancies and ensuring high efficacy rates, there is the need to understand the factors that predict their use among adolescent girls and young women in high fertility countries. We examined the predictors of *LARCs* use among adolescent girls and young women in high fertility countries in SSA.

## Methods

### Data source and study design

This study involved secondary data analysis of the most recent Demographic and Health Surveys (DHS) data which were collected using a cross-sectional study design. Data from the top ten high fertility countries in SSA [15] were pooled from the women’s file in each country for the study (Table 1). The most recent datasets of the ten countries dated between 2010 and 2020 were considered for inclusion in the study. As shown in the literature, DHS is a nationally representative study conducted in several LMICs across the globe [16]. The survey employed a two-stage sampling technique in recruiting respondents. Detailed sampling methodology has been highlighted in a previous study [17]. Structured questionnaires were used to collect the data from the respondents on health indicators such as contraceptive use [16]. A weighted sample of 5,854 adolescent girls and young women aged 15–24 was included in the final analysis. The survey dataset is freely available to download at https://dhsprogram.com/data/available-datasets.cfm. This manuscript was written per the Strengthening Reporting of Observational Studies in Epidemiology (STROBE) guidelines [18].

**Table 1:** Description of the sample

S/N Country	Year of survey	Weighted N	Weighted %
1. Burkina Faso	2010	740	12.69
2. Angola	2015–16	769	13.18
3. Burundi	2016–17	575	9.85
4. Gambia	2019–20	186	3.19
5. Mali	2018	470	8.05
6. Mozambique	2011	618	10.59
7. Nigeria	2018	739	12.66
8. Niger	2012	183	3.13
9. Chad	2014–15	156	2.68
10. Uganda	2016	1399	23.98
All countries	2010–2020	5834	100.00

### Variables

*LARCs* utilisation was the outcome variable in this study. To assess this variable, sexually active adolescent girls and young women were asked to indicate the type of contraceptive they were using. Those whose type of contraceptive fell outside the types of modern contraceptives were dropped. Among those using modern contraceptives, women who were using an intrauterine device (IUD) and implant were categorised as using *LARCs* and was coded as “1” whilst the remaining groups of modern contraceptives were coded as “0 = not using *LARCs*”. This categorisation of LARC was based on studies that used the DHS dataset [12, 13].

A total of twelve variables were included in the study as explanatory variables. These variables were further sectioned into individual level and contextual level variables. The individual level variables and their categorisation were as follows: age of the respondents (15–24), level of education (no formal education; primary; secondary or higher), marital status (never married; married; cohabiting; formerly married), current working status (not working; working), exposure to radio (no; yes), exposure to television (no; yes), exposure to newspaper or magazine (no; yes), desire for more children (wants more; wants no more; undecided); and parity (zero; one-three births; four or more births). Wealth index (poorest; poorer; middle; richer; richest), place of residence (urban; rural), and the 10 countries used for the study were the contextual level variables. The explanatory variables were selected based on their significant association with contraceptive use from literature as well as their availability in the DHS dataset [12, 13, 19–22].

### Statistical analyses

Statistical analysis was carried out using Stata version 16.0. Initially, the data were pooled from each of the 10 countries. Data cleaning and weighting were carried out in each country before appending them for the analysis. The weighting was carried out to obtain unbiased estimates of the results. After appending, the data were reset to a survey type using the surveyset (svy) command in Stata and the surveyset command was used throughout the analysis. Only the sample with complete observation for the variables of interest was included in the final analysis. Percentages were used to summarise the results of the prevalence of *LARCs* use, using a bar chart. Subsequently, cross-tabulations were performed to determine the distribution of *LARCs* use across the explanatory variables. A Pearson chi-square test of independence was later performed to examine the variables significantly associated with *LARCs* use. All significant variables were deemed qualified for inclusion into the regression model. Due to the complex data structure and the hierarchical nature of the DHS dataset, we adopted the multilevel regression model and this was carried out in a binary form. Four models were built to examine the predictors of *LARCs* use among adolescent girls and young women in the 10 countries. Model O (first model) was fitted to include only *LARCs*. The results from the model showed the variance of *LARCs* attributable to the clustering of the primary sampling (PSUs) without the explanatory variables used in the study. Model I, Model II, and Model III were fitted to include the individual-level variables, household/community level variables, and all the explanatory variables respectively. The results of the regression analysis were presented using adjusted odds ratio (aOR) with their respective 95% confidence intervals (CIs). Additionally, Akaike’s Information Criterion (AIC), an output of the random effect analysis was used to test for model fitness and for model comparison. The model with the least AIC was selected as the best-fitted model.

### Ethical consideration

Ethical clearance was not obtained for this study since the dataset is freely available in the public domain. Permission to use the DHS dataset was however sought from the MEASURE DHS after which approval was given. We complied with the ethical guidelines on the usage of secondary data for publication. The detailed information on the DHS data usage and ethical standards are available at http://goo.gl/ny8T6X.

## Results

**Fig. 1:**
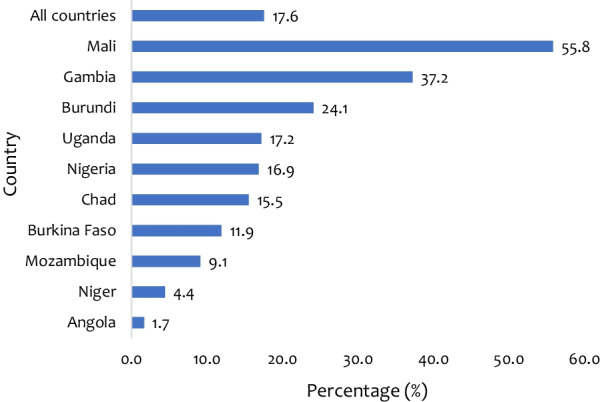
*LARCs* utilisation among adolescent girls and young women in high fertility countries in sub-Saharan Africa

Figure 1 shows the prevalence of *LARCs* utilisation in each of the 10 countries in SSA that participated in the study. The overall prevalence of *LARCs* utilization among adolescent girls and young women in the 10 countries was 17.6%. This varied from 1.66% in Angola to 55.8 in Mali.

Table 2 presents the results of the distribution of *LARCs* utilisation across the explanatory variables. The results indicate high proportion of *LARCs* utilisation among adolescent girls and young women aged 20–24 years (29.7%). With educational level, the highest prevalence of *LARCs* use (24.7%) was found among those with no education. It was also found that adolescent girls and young women who were married recorded high prevalence of *LARCs* utilisation (25.2%), whereas the lowest was observed among those who were never married (10.2%). High proportion of *LARCs* utilisation was also recorded among those currently working (19.4%). In terms of mass media, adolescent girls and young women who were not exposed to newspaper or magazine (19.2%), those not exposed to radio (18.7%) and those not exposed to television (20.2%) had a higher prevalence of *LARCs* utilisation. With parity and desire for more children, high proportion of *LARCs* use was observed among those with four or more births (25.7%), and those who do not want anymore children (23.0%). In terms of wealth index, the highest prevalence of *LARCs* utilisation (22.7%) was recorded among those in the poorest wealth quintile whereas those in the richest wealth quintile recorded the lowest (14.7%). With place of residence, those in the rural areas reported high prevalence of *LARCs* utilisation (20.9%). All selected independent variables were significantly associated with *LARCs* utilisation, except exposure to radio (see Table 2).

**Table 2:** Distribution of *LARCs* use across the explanatory variables

Variable	Weighted N	Weighted %	*LARCs* utilization	
			**Yes (%)**	**p-value**
Age of the respondents				
15–19	1641	28.1	12.1	< 0.001
20–24	4193	71.9	29.7	
Educational level				
No education	915	15.7	24.7	< 0.001
Primary	1737	29.8	18.8	
Secondary or higher	3182	54.5	14.8	
Marital status				
Never married	2116	36.3	10.2	< 0.001
Married	2014	34.5	25.2	
Cohabiting	1347	23.1	16.1	
Formerly married	357	6.1	23.4	
Current working status				
No	2515	43.1	15.2	< 0.001
Yes	3319	56.9	19.4	
Exposure to newspaper or magazine				
No	4983	85.4	19.2	< 0.001
Yes	851	14.6	8.1	
Exposure to radio				
No	2916	50.0	18.7	0.362
Yes	2918	50.0	16.4	
Exposure to television				
No	3205	54.9	20.2	< 0.001
Yes	2629	45.1	14.3	
Parity				
Zero birth	1741	29.8	5.6	< 0.001
One to three births	3922	67.2	22.5	
Four or more birth	171	2.9	25.7	
Desire for more children				
Wants more	5273	90.4	17.4	< 0.001
Wants no more	374	6.4	23.0	
Undecided	187	3.2	11.3	
Wealth index				
Poorest	539	9.2	22.7	< 0.001
Poorer	765	13.1	21.5	
Middle	958	16.4	19.2	
Richer	1366	23.4	16.7	
Richest	2206	37.8	14.7	
Place of residence				
Urban	2921	50.1	14.2	< 0.001
Rural	2913	49.9	20.9	

### Predictors of *LARCs*

Model III of Table 3 presents the results of the predictors of *LARCs* use among adolescent girls and young women in the 10 countries. The results revealed lower likelihood of *LARCs* utilisation among adolescent girls and young women who were married [AOR = 0.63, 95% CI = 0.45, 0.88] compared to those never married. In terms of desire for more children, it was discovered that adolescent girls and young women who wants no more children had higher odds of *LARCs* use [AOR = 1.56, 95% CI = 1.09, 2.26] compared to those who wanted more children. For parity, the study found that those with one to three births [AOR = 6.42, 95% CI = 4.27, 9.67] and those with four or more births [AOR = 7.02, 95% CI = 3.88, 12.67] were more likely to use LARCs relative to those who had no child. Countries in SSA with a lower probability of utilising LARCs in model III of Table 3 comprised Angola [AOR = 0.09, 95% CI = 0.04, 0.24], Niger [AOR = 0.23, 95% CI = 0.11, 0.50] and Mozambique [AOR = 0.53, 95% CI = 0.31, 0.92], whereas adolescent girls and young women in Mali [AOR = 12.22, 95% CI = 8.10, 18. 41] had higher probability of utilising LARCs compared to Burkina Faso (see Table 3).

**Table 3:** Fixed-random effects analysis of predictors of *LARCs* use among adolescent girls and young women

Variables	Model O	Model I AOR [95% CI]	Model II AOR [95% CI]	Model III AOR [95% CI]
Fixed-effect results				
Age of the respondents				
15–19		1 [1.00,1.00]		1 [1.00,1.00]
20–24		1.16 [0.92,1.45]		1.27 [0.99,1.64]
Educational level				
No education		1 [1.00,1.00]		1 [1.00,1.00]
Primary		0.73^*^ [0.55,0.97]		0.9 [0.67,1.23]
Secondary or higher		0.88 [0.67,1.15]		0.91 [0.67,1.23]
Marital status				
Never married		1 [1.00,1.00]		1 [1.00,1.00]
Married		0.96 [0.69,1.32]		0.63^**^ [0.45,0.88]
Cohabiting		0.65^**^ [0.47,0.89]		0.74 [0.53,1.04]
Formerly married		1.00 [0.63,1.59]		1.05 [0.67,1.65]
Current working status				
No		1 [1.00,1.00]		1 [1.00,1.00]
Yes		1.18 [0.98,1.42]		1.13 [0.92,1.39]
Exposure to reading newspaper or magazine				
No		1 [1.00,1.00]		1 [1.00,1.00]
Yes		0.53^***^ [0.36,0.77]		1.04 [0.70,1.55]
Exposure to listening to radio				
No		1 [1.00,1.00]		1 [1.00,1.00]
Yes		1.02 [0.83,1.24]		1.07 [0.86,1.34]
Exposure to watching television				
No		1 [1.00,1.00]		1 [1.00,1.00]
Yes		0.97 [0.79,1.21]		0.85 [0.66,1.11]
Desire for more children				
Wants more		1 [1.00,1.00]		1 [1.00,1.00]
Wants no more		1.21 [0.86,1.72]		1.56^*^ [1.09,2.26]
Undecided		0.55^*^ [0.32,0.96]		0.91 [0.51,1.64]
Parity				
Zero birth		1 [1.00,1.00]		1 [1.00,1.00]
One to three births		5.04^***^ [3.43,7.40]		6.42^***^ [4.27,9.67]
Four or more births		5.44^***^ [3.08,9.59]		7.02^***^ [3.88,12.67]
Wealth index				
Poorest			1 [1.00,1.00]	1 [1.00,1.00]
Poorer			0.97 [0.69,1.36]	0.95 [0.67,1.34]
Middle			0.88 [0.62,1.24]	0.92 [0.65,1.31]
Richer			0.80 [0.56,1.14]	0.91 [0.64,1.31]
Richest			0.83 [0.56,1.22]	1.09 [0.72,1.64]
Place of residence				
Urban			1 [1.00,1.00]	1 [1.00,1.00]
Rural			0.99 [0.75,1.32]	0.94 [0.69,1.28]
Country				
Burkina Faso			1 [1.00,1.00]	1 [1.00,1.00]
Angola			0.11^***^ [0.04,0.28]	0.09^***^ [0.04,0.24]
Burundi			2.28^***^ [1.52,3.42]	1.53^*^ [1.00,2.34]
Gambia			5.08^***^ [2.97,8.69]	3.76^***^ [2.19,6.48]
Mali			11.64^***^ [7.86,17.23]	12.22^***^ [8.10,18.41]
Mozambique			0.65 [0.40,1.05]	0.53^*^ [0.31,0.92]
Nigeria			1.43 [0.96,2.14]	1.85^**^ [1.21,2.84]
Niger			0.32^**^ [0.15,0.70]	0.23^***^ [0.11,0.50]
Chad			1.19 [0.56,2.52]	1.08 [0.50,2.33]
Uganda			1.43^*^ [1.03,2.01]	1.10 [0.76,1.59]
Random effect results				
PSU variance (95% CI)	0.93 [0.70, 1.24]	0.82 [0.60, 1.12]	0.84 [0.61, 1.15]	0.79 [0.57, 1.10]
ICC	0.22	0.20	0.20	0.19
Wald Chi-square	Reference	173.76***	335.52***	459.18***
Model fitness				
Log-likelihood	− 2657.37	− 2492.68	− 2340.47	− 2215.75
AIC	5818.74	5017.36	4712.94	4491.51
N	5834	5834	5834	5834
Number of clusters	954	954	954	954

Exponentiated coefficients; 95% confidence intervals in brackets; AOR = adjusted odds ratios; CI Confidence Interval; ^*^p < 0.05, ^**^p < 0.01, ^***^p < 0.001; 1 [1.00,1.00] = Reference category; PSU = Primary Sampling Unit; ICC = Intra-Class Correlation; AIC = Akaike’s Information Criterion

## Discussion

The purpose of this study was to examine the prevalence and predictors of *LARCs* utilisation among adolescent girls and young women in ten countries in SSA. The study revealed that the overall prevalence of *LARCs* utilisation is 17.6%. This varied from 1.7% in Angola to 55.8% in Mali. This indicates that the vast majority of adolescent girls and young women in these countries did not use LARCs. Our findings are consistent with prior studies in Kenya [14] and Ethiopia [23–25]. The consistency of these findings could be explained by the study's population characteristics, as the majority of adolescent girls and young women chose short contraceptive methods over the *LARCs*. However, the findings of this study are higher than other studies in Tanzania [26], North Ethiopia [27] and Guatemala [28]. It is possible that the use of LARCs in the study group has only lately risen. It had earlier not been recommended for this age group since they had not yet reached their preferred family size. Nevertheless, *LARCs* has currently been suggested and encouraged for adolescent girls and young women with no children, hence the rise in usage [14, 29, 30]. The result is lower than that of a study of sexually active women from 26 countries in SSA, which found that 21.73% of them use *LARCs* [12]. The rise in the use of LARCs could be attributed to enhanced community mobilisation and understanding of LARCs among women, as well as variations in study time, location, and socio-demographic backgrounds [31, 32].

The current study found marital status, desire for more children, and parity to be significantly associated with *LARCs* utilisation. Adolescent girls and young women who were married had lower odds of using LARCs compared to those who were never married. This finding is in line with prior studies that have showed negative relationship between marriage and current contraceptive utilisation [33, 34]. As indicated in the study at Democratic Republic of Congo, a greater percentage of married women were less likely to use modern procedures and LARCs for pregnancy prevention [34]. This could be due to pressure from family or society to have a child shortly after marriage [34]. Adolescent girls and young women should therefore be encouraged to use contraception during antenatal care contacts in order to hearten child spacing and postpone pregnancies. Our findings, however, contradict prior studies in Ghana [35] and Nigeria [12, 36] which showed that married women were more likely to use modern contraception for child spacing and limiting births. A probable explanation may be that adult mothers seem to have more children, have reached their family size limit, and do not want any more children, therefore preferring the *LARCs* technique over short-acting contraceptives [13].

Desire for more children was revealed to have a statistically significant association with *LARCs* use among adolescent girls and young women in SSA. When compared to adolescent girls and young women who had desire for more children, those who had no desire for more children showed higher likelihood of *LARCs* utilisation. Thus, we discovered that adolescent girls and young women who did not want more children utilised *LARCs* more frequently, implying that they are employing procedures that fit their fertility goals. The number of living children was found to have a significant favorable impact on the utilisation of *LARCs* [14]. Our finding corroborates prior studies [13, 20, 37] which revealed that adolescent girls and young women who showed no desire for more children were more likely to use LARCs to prevent pregnancy. This is to be expected, as long-acting contraceptives are widely regarded as everlasting contraceptive techniques and are primarily used to prevent pregnancy [38]. One possibility is that these adolescent girls and young women had already achieved their reproductive goals.

Our study also discovered that *LARCs* utilisation was higher among adolescent girls and young women who had at least one child compared to adolescents and young women with no children. The likelihood of using a *LARCs* increases as the number of children rises [39]. This finding is in congruent with other studies [12, 13, 39–41], where parity was found to be significantly connected with individuals’ fertility behaviours, including request and utilisation of contemporary contraception. This conclusion could be explained by the fact that multiparous women are more likely to get family planning information and contraceptive usage counseling during their pregnancy period, boosting their chances of utilising LARCs [39]. Additionally, a woman with a higher parity may be exposed to several contraception information and experience throughout antenatal and postnatal contacts [39, 42]. The fact that the likelihood of using *LARCs* increased as the number of children increased could indicate that family planning/maternal child health programs are effectively integrated, because when adolescent girls and young women use these services, they are exposed to more *LARCs* information and services and can consider meeting their request for spacing or restricting children [14].

In our analysis, adolescent girls and young women in countries, including Angola, Niger, and Mozambique were shown to have a reduced likelihood of using LARCs, whereas adolescent girls and young women in Mali had a higher likelihood of using LARCs. This could be due to the fact that Mali has programs in place to encourage adolescents and young women to use *LARCs*. The Malian government and family planning professionals’ good efforts to promote modern contraception have resulted in increased *LARCs* utilisation [43, 44].

### Strengths and limitations

The study’s main strength is its relatively large sample size, which allows the findings to be applied to all adolescent girls and young women in the countries that participated in this study. A limitation of this study is that due to the cross-sectional nature of the survey we could only draw associations but not causality among the studied variables. Additionally, due to economic, cultural, and social disparities among countries in SSA, the various recommendations suggested in this study may not be relevant in all sub-Saharan African countries. Furthermore, the impacts of *LARCs* accessibility and adolescent girls and young women's views about *LARCs* use were not taken into account in this study.

## Conclusions

The use of LARCs was found to be relatively low in the ten countries included in this study. To improve the use of *LARCs* among adolescent girls and young women, it is important to implement various strategies and strengthen existing interventions taking into consideration the factors identified in this study.

## Data Availability

Dataset is freely available at https://dhsprogram.com/data/dataset.

## Abbreviations

COR: Crude odds ratios
AOR: Adjusted odds ratio
CI: Confidence interval
LARCs: Long-acting reversible contraceptives
AGYW: Adolescent girls and young women
SSA: Sub-Saharan Africa
SDG: Sustainable development goal
LMIC: Lower middle-income country
PRB: Population Reference Bureau
DHS: Demographic and Health Surveys
IUD: Intrauterine device
AIC: Akaike’s information criterion
ICC: Intra-class correlation
PSU: Primary sampling unit
